# Proteins related to lipoprotein profile were identified using a pharmaco-proteomic approach as markers for growth response to growth hormone (GH) treatment in short prepubertal children

**DOI:** 10.1186/1477-5956-7-40

**Published:** 2009-11-02

**Authors:** Björn Andersson, Gunnel Hellgren, Andreas FM Nierop, Ze'ev Hochberg, Kerstin Albertsson-Wikland

**Affiliations:** 1 Göteborg Pediatric Growth Research Center, Department of Pediatrics, Institute of Clinical Sciences, Sahlgrenska Academy, University of Gothenburg, Sweden; 2 Muvara bv, Leiderdorp, The Netherlands; 3 Meyer Children's Hospital, Rambam Medical Center and Rappaport Faculty of Medicine and Research Institute, Technion - Israel Institute of Technology, Haifa, Israel

## Abstract

**Background:**

The broad range in growth observed in response to growth hormone (GH) treatment is mainly caused by individual variations in both GH secretion and GH sensitivity. Individual GH responsiveness can be estimated using evidence-based models that predict the response to GH treatment; however, these models can be improved. High-throughput proteomics techniques can be used to identify proteins that may potentially be used as variables in such models in order to improve their predictive ability. Previously we have reported that proteomic analyses can identify biomarkers that discriminate between short prepubertal children with idiopathic short stature (ISS) who show good or poor growth in response to GH treatment. In this study we used a pharmaco-proteomic approach to identify novel factors that correlate with the growth response to GH treatment in prepubertal children who are short due to GH deficiency or ISS. The study included 128 short prepubertal children receiving GH treatment, of whom 39 were GH-deficient and 89 had ISS. Serum protein expression profiles at study start and after 1 year of GH treatment were analyzed using SELDI-TOF. Cross-validated regression and random permutation analyses were performed to identify significant correlations between protein expression patterns and the 2-year growth response to GH treatment.

**Results:**

At start of treatment we identified a combination of seven protein peaks that correlated with the 2-year growth response in the GH-deficient group (R^2 ^= 0.73). After 1 year of treatment, a combination of four peaks in the GH-deficient group (R^2 ^= 0.64), eight peaks in the ISS group R^2 ^= 0.47) and eight peaks in the total study group correlated with the 2-year growth response R^2 ^= 0.38).

The peaks identified corresponded to apolipoproteins A-I, A-II, C-I, C-III, transthyretin and serum amyloid A 4, which are all part of the high-density lipoprotein.

**Conclusion:**

Using a proteomic approach we identified biomarkers related to the lipoprotein profile that could be used to predict growth response to GH treatment in prepubertal children who are short as a result of GH-deficiency or who have ISS.

These results support our previous findings that apolipoproteins and transthyretin may have a role in GH sensitivity.

## Background

Growth during childhood depends, among other things, on the balance between the level of endogenously secreted growth hormone (GH) and the responsiveness of the target tissue to GH. Furthermore, a broad range of serum GH levels has been observed in children with similar growth rates [1] and it is known that GH exerts its stimulatory effect on growth in children during childhood in a dose-dependent way [2]. There is also considerable intra-individual variability in growth in response to GH treatment among children who are GH-deficient and among those who have idiopathic short stature (ISS) [3-6]. To deal with this complexity, we and others have constructed evidence-based models for predicting growth in response to GH treatment [7-11]. These models provide an indirect measurement of individual responsiveness to GH [12]. The best models available today explain up to 80% of the growth in response to GH. Early growth data, auxological data of the child and the parents and the level of spontaneous GH secretion over 24 h are important variables in these models [7-11]. Because some of these parameters, such as early growth data and parental auxological data, are not always readily available, there is a need to develop a model that includes only parameters that can be obtained at the start of the growth investigation at the pediatric unit. To achieve this it is necessary to change the focus from single marker studies toward a broader search for multiple markers of growth response using high-throughput techniques.

We have previously used surface-enhanced laser desorption/ionization time-of-flight mass spectrometry (SELDI-TOF MS) to identify biomarkers that discriminate between good and poor responders to GH treatment among a group of children with ISS [13]. We showed that information on the change in peak intensities of apolipoprotein (Apo) A-II and transthyretin (TTR) during the first year of GH treatment could be used to correct classify 82% of children receiving GH as good or poor treatment responders, respectively [13].

In this study we used the same technique to search for biomarkers that correlated with growth response to GH treatment in short prepubertal children, who were either GH-deficient or of ISS. Serum samples taken at the start of a clinical trial of GH and after 1 year of treatment from children with a broad range of levels of GH secretion at start were analyzed. We found that serum markers related to nutrition and fat transport in the body correlated with the 2-year growth response.

## Subjects and Methods

### Ethical consideration

The protocol was approved by the ethical boards of the Universities of Gothenburg (for patients from Gothenburg and Halmstad), Umeå, Uppsala and Malmö and the Medical Product Agency of Sweden. Written informed consent was obtained from all parents and from children if old enough. The trial was performed in accordance with the Declaration of Helsinki and Good Clinical Practice guidelines.

### Study population

The per-protocol study population from the GH dose clinical trial (TRN 98-0198-003) consists of 128 short prepubertal children of Caucasian origin receiving GH treatment; see [5] for more detailed information. Study patients were randomized either to a group receiving an individualized (two-thirds of patients) or a standard GH dose (one-third of patients). The standard GH dose was 43 μg/kg/day. The individualized GH dose comprised one of six different doses (mean 49, range 17-100 μg/kg/day), calculated using a prediction model that considered estimated GH sensitivity and the difference between the current height of the child and mid-parental height (MPH), as previously described [12].

The maximum peak GH secretion (GH_max_) ≥ 32 mU/L on an arginine-insulin tolerance test (AITT) or of the spontaneous GH secretion over a 24 h period was used to classify the patients as having either ISS (n = 89) or short stature due to GH deficiency (n = 39). Clinical data for the patient groups are presented in Table 1.

**Table 1:** Auxiological data for the study groups

**A) Total group (n = 128; 38 girls, 90 boys)**				**B) ISS (n = 89, 25 girls, 64 boys)**			**C) GHD (n = 39, 13 girls, 26 boys)**		
**Variables**	**Median**	**Min**	**Max**	**Median**	**Min**	**Max**	**Median**	**Min**	**Max**

**At birth**									

Gestational age (weeks)	39	33	42	39	33	42	39	36	42

Height SDS	-0,66	-2,52	1,68	-0,74	-2,52	1,68	-0,70	-2,52	1,68

**At GH start**									

Age (yrs)	7,27	3,05	10,95	7,32	3,24	10,95	7,14	3,05	10,94

Height SDS	-2,64	-4,10	-1,77	-2,64	-4,10	-1,77	-2,59	-3,65	-1,98

Weight SDS	-2,24	-4,31	0,26	-2,39	-4,31	-0,45	-2,03	-3,80	0,26

BMI SDS	-0,47	-3,35	2,11	-0,55	-3,35	1,88	-0,20	-1,80	2,11

GH_max _AITT (mU/L)	16,95	1,10	62,90	19,60	1,10	62,90	15,10	1,60	22,30

GH_max _24 h (mU/L)	24,85	0,80	58,10	28,10	12,70	58,10	14,70	0,80	22,10

Diff MPHSDS	-1,73	-3,53	-0,61	-1,72	-3,53	-0,61	-1,77	-2,45	-0,78

HDL (mg/L)	1,44	0,64	2,70	1,44	0,64	2,70	1,44	0,91	2,24

IGF-I SDS	-1,10	-5,17	2,25	-1,19	-3,84	2,25	-1,06	-5,17	0,91

GH dose (μg/kg/day)	44,20	17,00	102,00	44,20	17,00	102,00	44,20	17,00	68,00

**After 2 yrs of treatment**									

Height SDS	-1,45	-3,00	0,20	-1,45	-3,00	0,20	-1,45	-2,42	0,05

Delta height SDS 2 yrs	1,26	0,23	2,69	1,22	0,23	2,69	1,31	0,51	2,39

BMI SDS	-0,24	-3,10	2,03	-0,05	-3,10	1,88	-0,08	-2,14	2,03

HDL (mg/L)	1,36	0,76	2,06	1,37	0,76	2,06	1,28	0,94	1,97

IGF-I SDS	1,57	-1,48	4,21	1,49	-0,65	3,82	1,57	-1,48	4,21

AITT, arginine--insulin tolerance test; GH_max_, maximum peak of GH secretion; IGF-I, insulin-like growth factor I; GHD, GH deficient; MPH, mid-parental height; diffMPHSDS, difference in height SDS of the child versus its mid-parental height SDS

0.1 U GH/kg/day = 33 μg/kg/day

The reference values used for SDS calculations were obtained from [18] for height and weight and [15] for IGF-I.

### Study design

Fasting blood samples were taken at the start of the study and after 1 year on GH treatment. Samples were stored at -70°C and were not thawed until the time of analysis. No sample was stored for more than 8 years before analysis.

### Hormone evaluation

Published reference values were used to assess the results of analyses of GH [14], insulin-like growth factor I (IGF-I) [15] and IGF-binding protein 3 (IGFBP-3) [16], which were performed at the GP-GRC laboratory (Swedac accredited no 1899) at the University of Gothenburg. High-density lipoprotein (HDL) was measured at the Department of Clinical Chemistry, Sahlgrenska University Hospital (accredited according to the international standard ISO/IEC 17025).

### Growth evaluation

The childhood component [17] of the Swedish population-based growth reference values was used for the height-related inclusion criteria and to express the height, weight [18] and body mass index [19] of the patients and their parents. Reference standards of newborns were used for standard deviation score (SDS) at birth [20].

### Surface-enhanced laser desorption/Ionization time-of-flight mass spectrometry (SELDI-TOF MS) serum protein profiling

Serum samples were thawed, denatured and fractionated using anion-exchange beads in a serum fractionation kit (Bio-Rad Laboratories, Hercules, CA) according to protocols provided by Bio-Rad Laboratories. Based on results from a previous study [13], serum fraction 5 and 6 (pH 3 and organic solvent) were analyzed together using weak anion-exchange (CM10) arrays, fraction 1 (flow through) was analyzed by immobilized metal-affinity capture (IMAC30) arrays and fraction 4 (pH 4) was analyzed using reversed-phase (H50) arrays. CM10 arrays were equilibrated twice with 150 μl binding buffer (100 mM NaAcetate, pH 4.0). IMAC30 arrays were charged using 50 μl 0.1 M CuSO4, 10 min, washed with 150 μl H_2_O, 1 min, neutralized with 150 μl 0.1 M NaAc pH 4.0, 5 min, washed with 150 μl H_2_O, 1 min, equilibrated twice with 150 μl binding buffer (0.1 M Na3PO4, 0.5 M NaCl, pH 7.0). H50 arrays were washed with 200 μl 50% acetonitrile (ACN) (Merck, Darmstadt, Germany) 2 × 5 min, equilibrated twice with 150 μl binding buffer (10% ACN, 0.1% trifluoroacetic acid (Merck)). After equilibration, a 10 μl sample and 90 μl binding buffer were applied to duplicate samples on the arrays and mixed at room temperature using a DPC MicroMix 4 for 1 h (CM10 and IMAC30) or 1.5 h (H50). After protein binding, the arrays were washed three times with 150 μl binding buffer, rinsed twice with 150 μl 1 mM HEPES, and air-dried. Afterwards, 0.6 μl of a 50% solution of sinapinic acid (SPA) (Bio-Rad Laboratories) in 0.5% trifluoroacetic acid and 50% ACN were applied twice to each spot as a matrix.

Time-of-flight spectra were generated using a PBS IIc ProteinChip reader (Bio-Rad Laboratories). Instrument settings for the analysis were optimized in the mass range of 2.3-20.0 kDa and data were averaged from 180 transients for each protocol. To minimize experimental variation, all samples were randomized and analyzed concurrently within 1 week by the same operator. In addition, one reference serum sample was randomly applied on each array and evaluated. The mass accuracy was calibrated in the molecular range of 5-18 kDa using external calibrators from Bio-Rad Laboratories. The same calibration equation was used for all samples.

### Data preprocessing

Data handling was performed using ProteinChip Data Manager (Bio-Rad Laboratories). All spectra were baseline-subtracted and normalized according to total ion current. Settings for peak identification and clustering of peaks across multiple spectra were first pass signal-to-noise ratio (S/N) > 3 in 15% of all spectra and second pass S/N > 2, with a cluster mass window of 0.3% of the mass.

Spectra were visually inspected and patients were excluded from further data analysis if profiles clearly differed between the duplicate samples or if the overall quality was low in one or both of the spectra (i.e. high noise, overall low peak intensity or an abnormal normalization factor in combination with visually deviating spectra). This process resulted in the identification of 147 valid peaks for CM10 (n = 67), IMAC30 (n = 46) and H50 (n = 34) in the mass/charge (m/z) area between 2.3 and 30.0 kDa. The average coefficient of variations (CV) for the peaks detected in all of the reference samples was 30.1% for CM10, 34.1% for IMAC30 and 32.8% for H50.

Only patients for whom there were two high-quality mass spectra for the relevant array and time point were included in further statistical analysis. This resulted in a study population of 128 children for the CM10 analyses at all time points. For analyses of peak data from the three different arrays merged together, the study populations were 121 children at the start of the study, 124 children after 1 year and 120 children for the change between baseline and 1 year (delta 0-1 year).

### Protein identification

ACN precipitation was performed, as previously described [21], on the pooled fraction 5 and 6 to remove high molecular weight proteins. The precipitate was subjected to SDS page and Coomassie blue staining to visualize the proteins. The protein bands with molecular weights corresponding to the biomarkers of interest were cut out and passive elution was performed. First the excized gel pieces were washed with 50% ACN/50 mM Ambic for 3 × 15 min or until the gel pieces were destained. The gel pieces were dehydrated with 100% ACN, heated to 50°C for 5 min and thereafter dried in a Speed-Vac. 100 μl of 45% formic acid, 30% ACN and 10% isopropanol was added. The tubes were sonicated for 30 min in a water bath at room temperature and incubated at room temperature for approximately 4 h. One microliter of each sample was analyzed on a NP20 ProteinChip array with saturated SPA. The remainder of the sample obtained from passive elution was incubated overnight and sonicated the next morning. Each supernatant was transferred to a new tube and dried in a Speed-Vac. Depletion experiments and in-gel digestions were performed as previously described [13]. Protein identification by nanoflow LC-MS/MS was performed on a hybrid linear ion-trap Fourier transform ion cyclotron resonance (FTICR) mass spectrometer (LTQ-FT, Thermo Electron, Bremen, Germany), as previously described [13].

### Statistics

For all analyses the 2-year growth response (delta height SDS 0-2 years) [7] was used as the outcome variable. All peaks (n = 147) detected on the different surfaces were merged and analyzed together. In addition, each surface was analyzed individually. The peak intensity data were analyzed both directly after pre-processing and after transformation to a logarithmic scale. Serum protein profiles were analyzed before and after 1 year of treatment, and in terms of the change in profiles over 1 year of treatment.

#### Multivariate statistics

Multivariate data analysis was performed with Matlab software (version 7.7.0 R2008b, The Mathworks) on the mean intensity levels of the duplicate samples. Cross-validated stepwise regression was computed to find subsets of peaks that correlated with the delta height SDS 0-2 years. Final selection of reliable subsets of predictive peaks was based on a random permutation test. The identified peaks were analyzed thereafter using multidimensional scaling (MDS) to explore the relationships between the peaks.

#### Between-duplicate variation

To estimate the reliability of the peaks compared with their biological range, the ratio of the between-duplicate variation and the total variation was computed, giving the proportion of variance explained by duplicates. A low value for a certain peak meant that there was relatively little variation between the duplicates compared with the total expected biological and instrumental variation.

#### Cross-validated stepwise regression

Using stepwise regression, subsets of peaks were selected with leave-one-out cross-validation to examine the correlation of the peaks with delta height SDS 0-2 years. Sets of potential regression models were generated using between 1 and a maximum of 15 peaks.

#### Random permutation tests

To study the robustness of the data analyses, random permutation tests were performed on the complete stepwise regression procedure described above including the selection of subsets of peaks based on the highest cross-validated R^2^. For each number of peaks, we tested for 999 permutations if the permuted cross-validated R^2 ^was equal to or above 90% of the calculated true cross-validated R^2^. In other words, we assessed if there was a significant gap (10%) between the calculated true cross-validated R^2 ^and the distribution of all permuted cross-validated R^2^. Random permutation tests resulting in a p-value < 0.05 were considered significant. For each number of peaks, the best regression model was selected based on a significant p-value in the permutation tests, in combination with a relatively low number of peaks in the regression model and a relative high cross-validated R^2^.

#### Analysis of systematic errors

Stepwise regression was used to analyze the impact of systematic errors on the results. No systematic errors were found.

## Results

### Protein expression pattern

Data from the spectra generated were analyzed both as merged peak data from all analyzed surfaces and in terms of each individual surface. Best results, with respect to the lowest permutation test p-value in combination with high cross-validated R^2^, were obtained using data from only CM10 on pooled fractions 5 and 6. All data were analyzed for the GH-deficient group, the ISS group and for the total group.

### At start of GH treatment

**Figure 1:**
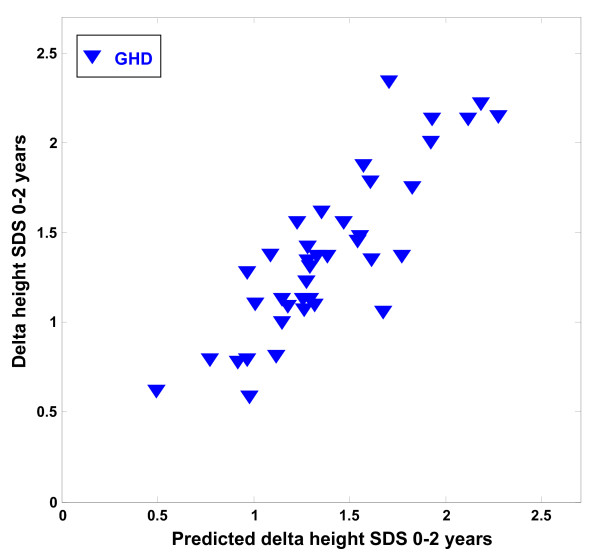
**Protein peaks at treatment start that were predictive of 2-year growth response in GH-deficient children**. The figure shows the correlation between the observed delta height SDS 0--2 year (y-axis) and the predicted delta height SDS 0--2 year (x-axis), using the combined intensities of the predictors with M/Z values --9135, +3318, +17390 (Apo A-II), +8767 (Apo A-II), +12872 (SAA 4), +9642 (Apo C-III), +3160 (r = 0.73, p = 0.032), in the GH-deficient children (GHD) at start of treatment. A positive sign indicates a positive correlation with the outcome variable whereas a negative sign indicates a negative correlation with the outcome variable.

In the GH-deficient group of children we identified a specific protein expression pattern of seven peaks that correlated with the delta height SDS 0-2 years (R^2 ^= 0.73, p = 0.032) (Table 2). The correlation between the predicted and the observed delta height SDS 0-2 years is shown in Figure 1. No significant correlations between the protein expression pattern and the delta height SDS 0-2 years were identified for the total group of patients or the ISS group at start of treatment.

**Table 2:** The most predictive peaks for delta height_SDS _0-2 year identified by regression analysis

**Group**	**Patients (n=)**	**Peaks (n=)**	**R**^2^	**Cross-validated R**^2^	**p-value**	**Peak m/z value (kDa)**
GHD	39	7	0,73	0,61	0,032	3.160, 3.318, 8.767 (Apo A-II), 9.135, 9.642, 12.872 (TTR), 17.390 (Apo A-II)
GHD	39	4	0,64	0,53	0,017	4.408 (Apo A-II), 8.696 (Apo A-II), 9.019 (Apo A-II), 17.146 (Apo A-II)
ISS	89	8	0,47	0,35	0,015	3.160, 4.470, 6.857 (Apo C-I), 8.767 (Apo A-II), 8.875 (Apo A-II), 9.425 (Apo C-III), 12.607 (SAA4), 12.872 (TTR)
Total	128	8	0,38	0,28	0,003	4.628, 4.470, 4.793, 8.817, 8.875, 9.019, 12.872, 17.146
GHD	39	4	0,59	0,48	0,026	4.138, 8.817 (Apo A-II), 9.019 (Apo A-II), 17.262 (Apo A-II)
Total	128	8	0,35	0,24	0,003	4.138, 8.636 (Apo A-II), 8.875 (Apo A-II), 9.135, 9.425 (Apo C-III), 14.055 (TTR), 28.090 (Apo A-I), 29.003

GHD, GH-deficient; ISS, idiopathic short stature.

The most predictive peaks identified by regression analysis at start, after 1 year, and during the first year of GH treatment. For each model, the R^2^-value and cross-validated R^2 ^are presented.

### After 1 year of GH treatment

**Figure 2:**
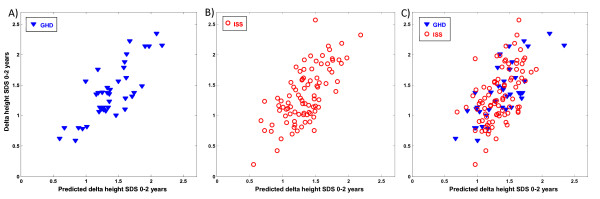
**Protein peaks at 1 year of treatment that were predictive of 2-year growth response**. The figure shows the correlation between the observed delta height SDS 0--2 year (y-axis) and the predicted delta height SDS 0--2 year (x-axis), using the combined intensities of the predictors with M/Z values A) --17146 (Apo A-II), +8696 (Apo A-II), --9019 (Apo A-II), +4408 (Apo A-II) (r = 0.64, p = 0.017). B) +4470 (Apo A-II), --8767 (Apo A-II), --12872 (TTR), --12607 (SAA 4), +8875 (Apo A-II), +9425 (Apo C-III), --3160, --6857 (Apo C-I) (r = 0.47, p = 0.015). C) --4628, +4470 (Apo A-II), +8817 (Apo A-II), +4793, --17146 (Apo A-II), --12872 (TTR), --9019 (Apo A-II), +8875 (Apo A-II) (r = 0.38, p = 0.003) after 1 year of treatment in (A) GH-deficient children (GHD), (B) children with idiopathic short stature (ISS), and (C) the total group. A positive sign indicates a positive correlation with the outcome variable whereas a negative sign indicates a negative correlation with the outcome variable.

In the GH-deficient group, a protein expression pattern of four peaks correlated with the delta height SDS 0-2 years (R^2 ^= 0.64, p = 0.017) (Table 2, Figure 2A). In the ISS group, a protein expression pattern of eight peaks correlated with the delta height SDS 0-2 years (R^2 ^= 0.47, p = 0.015) (Table 2, Figure 2B). In the total group of children, the expression pattern of eight peaks correlated with the delta height SDS 0-2 years (R^2 ^= 0.38, p = 0.003) (Table 2, Figure 2C).

### During 1 year of GH treatment

**Figure 3:**
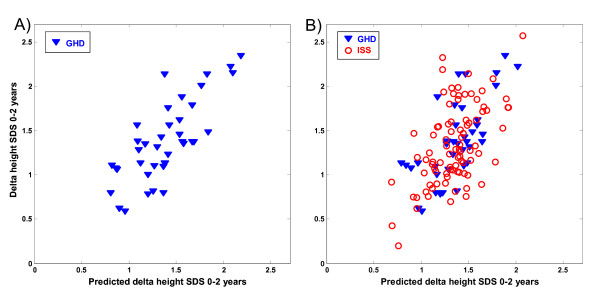
**Protein peaks during the first treatment year that were predictive of 2-year growth response**. The figure shows the correlation between the observed delta height SDS 0--2 year (y-axis) and the predicted delta height SDS 0--2 year (x-axis) in (A) GH-deficient children (GHD) and (B) the total group, using the combined changes in intensities of the predictors with M/Z values A) +Dy8817 (Apo A-II), +Dy4138, --Dy9019, --Dy17262 (Apo A-II) (r = 0.59, p = 0.026). B) +Dy4138, --Dy28090 (Apo A-I), +Dy14055 (TTR), +Dy8875 (Apo A-II), +Dy9135, +Dy29003, --Dy9425 (Apo C-III), --Dy8636 (Apo A-II) (r = 0.35, p = 0.003). A positive sign indicates a positive correlation with the outcome variable whereas a negative sign indicates a negative correlation with the outcome variable.

Finally we assessed correlations between changes in peaks intensities during the first year of GH treatment and the growth response after 2 years of treatment. In the GH-deficient group, the change in expression pattern of four specific peaks correlated with delta height SDS 0-2 years (R^2 ^= 0.59, p = 0.026) (Table 2, Figure 3A). There were no significant correlations found involving the ISS group. For the total group, the change in expression pattern of eight specific peaks correlated with delta height SDS 0-2 years (R^2 ^= 0.35, p = 0.003) (Table 2, Figure 3B).

### Peak identification

The protein expression patterns that provided the best predictive peaks for the 2-year growth response in the GH-deficient group, the ISS group and the total group, included a total of 23 unique peaks (Table 2). To identify the proteins corresponding to the peaks of interest we used the consistency of the peak pattern in the spectra, MS identification and serum depletion experiments.

#### Consistency of peak pattern in spectra

From the consistency of the peak patterns in the spectra, the peaks with m/z values around 14 kDa were recognized as different post-translational modified forms of TTR; the 14.055 kDa peak was recognized as the cysteinylated form and the 12.872 kDa peak as a truncated form. The 17.146, 17.262 and 17.390 kDa peaks were recognized as dimers of Apo A-II, and the 8.636 and 4.408 kDa peaks were recognized as truncated forms of Apo A-II. The 28.090 peak was recognized as Apo A-I.

#### MS protein identification

All peaks of interest were analyzed using MS protein identification. MS protein identification verified that the 4.408 and 4.470 kDa peak represented Apo A-II. In addition, the cluster of peaks between 8.636 and 9.019 kDa were identified as Apo A-II. The 6.857 kDa peak was identified as Apo C-I. The MS identification result for the 9.425 kDa peak indicated that this sample was not pure. However, based on the Mascot search result score (score: 2073, number of assigned peptides 51), this peak most likely represented Apo C-III. However Apo A-I was also present in the sample, but with a lower Mascot search result score (score 352, number of assigned peptides 29). The 12.607 kDa peak was identified as serum amyloid A 4 (SAA 4). The identity of the remaining peaks could not be determined accurately using MS.

#### Depletion experiments

**Figure 4:**
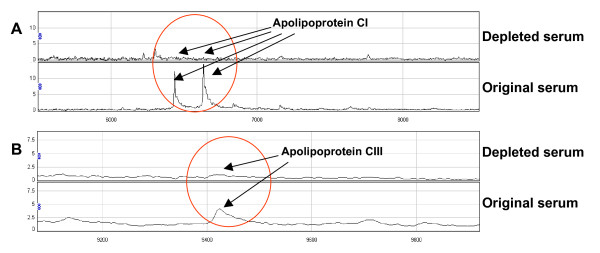
**Depletion experiment identifying (A) apolipoprotein C-I, and (B) apolipoprotein C-III**. (A) Anti-apolipoprotein C-I antibody depleted the 6.6 and 6.8 kDa peaks. (B) Anti-apolipoprotein C-III antibody depleted the 9.4 kDa peak. In both parts of the figure, the top panel shows the depleted serum and the bottom panel shows the original serum.

To verify the identities obtained from MS analyses of the proteins, depletion experiments using specific antibodies were performed. The depletion experiments using anti-Apo C-I and anti-Apo C-III antibodies (Abnova, Taipei City, Taiwan) confirmed that the 6.857 peak represented Apo C-I (Figure 4A) and the 9.425 kDa peak represented Apo C-III (Figure 4B). The 14.055 kDa peak has previously been confirmed to represent TTR [13].

### Peak intensity changes

The change in absolute peak intensities during the first year of GH treatment for the Apo A-I, Apo A-II, Apo C-I, Apo C-III, TTR and SAA 4 proteins found in this study were fairly small. Apo A-II and SAA4 are significantly decreased (p= < 0.00001 and p = 0.039, respectively) while Apo C-I and Apo C-III are significantly increased (p = 0.025 and p = 0.001, respectively). Apo A-I and TTR remained unchanged.

## Discussion

In this study we have identified serum protein profiles that correlated with the 2-year growth response to GH treatment in prepubertal children with GH deficiency and ISS. By using a combination of the specific peak patterns within the spectra, MS identification, and serum depletion experiments, proteins representing a subset of peaks within the profiles were identified. The majority of the proteins identified represent different apolipoproteins; Apo A-I, Apo A-II, Apo C-I and Apo C-III. Other proteins identified were TTR and SAA 4. These results support previous data suggesting that Apo A-II and TTR may have a role in determining GH sensitivity. The change in intensity of these peaks has been shown to allow the classification of children with ISS as good or poor responders to GH treatment [13].

All proteins identified in the current study are part of the HDL [22-24], but Apo A-II, Apo C-I, Apo C-III and SAA 4 have also been found in very low-density lipoproteins and low-density lipoproteins (LDLs) [24,25]. HDL is sometimes called 'good' cholesterol as it binds cholesterol and transports it to the liver. It is believed that HDL can remove cholesterol from atheroma within arteries and transport it back to the liver for excretion or re-utilization [26]. Cholesterol contained in HDL particles, unlike cholesterol within LDL particles, is considered beneficial for maintaining cardiovascular health. Today, not much is known about the effects of GH on either the apolipoproteins or on TTR and SAA 4. There are contradictory results regarding the effects of GH treatment given as daily subcutaneous injection on the HDL which carries these proteins [27-29]. In the present study we found that HDL slightly but significantly decreased during the first year of treatment (data not shown) in contrast to one study that shows increased levels of HDL in only the prepubertal group of boys of whom one third went into puberty during the third year follow-up period [28] Two studies showed almost unchanged levels, one in pubertal GH-deficient patients [27] and one in young adults [29]. In adults, it has been shown that a frequent low GH dose, which gave rise to an almost constant level of plasma GH [30], increased HDL. In contrast, an HDL-lowering effect was seen with a high single GH dose which created a GH plasma profile with an high peak after the GH injection that gradually decreased towards the next daily injection [29,31]. This may partly explain the decrease in HDL seen in our study because the children were given a single daily dose of subcutaneous GH resulting in a plasma pattern of GH with an initial peak and undetectably low levels of GH before next injection [32]. This pattern is more similar to the male GH secretion pattern with a high peak during the night, than the female secretion pattern with uniform GH secretion during both the day and night [33] It is well known that this gender specific secretion pattern is the signal for different growth [34] and metabolic effects, not least in the liver, in male and female rats [35,36]. Furthermore, GH affects lipolysis in the body [34] and by doing so probably shifts the energy balance in the body to a more optimal one for longitudinal growth.

From this study it is not possible to draw conclusions as to whether the markers identified are actually involved in the regulation of longitudinal growth or if they are indirect markers of the effects of GH on HDL levels during treatment. The different levels of the identified proteins may be a consequence of the altered levels of HDL and changes in the homeostasis of the lipoproteins. Interpretation of the results is also complicated by the presence of different regulated isoforms and cleavage products of Apo A-II as described in the legends to Figures 1, 2 and 3. The physiological significance of the presence of this variety of isoforms/cleavage products should be investigated in future studies.

On the target tissue level one can say that both the GH-deficient and the ISS child are GH-deficient; the deficient one due to low levels of secreted GH, and the idiopathic short child due to GH insensitivity in the target tissue, which often can be overcome by a higher dose of GH treatment [3]. The underlying reason for the tissue insensitivity can vary, giving rise to different phenotypes, whereas the GH-deficient children are of a more similar phenotype. However, there is no clear cut-off for GH secretion between these groups. In this study, different protein patterns were found to correlate with growth in response to treatment for the GH-deficient children and the children with ISS. At the start of treatment, we could only identify specific serum profiles correlating with growth response in the GH-deficient group of patients. There were strong correlations between the 1- and 2-year growth responses in all groups. Interestingly, there was no overlap between the peaks included in the models for the GH-deficient and the ISS groups. In the model for the total group, peaks from both the GH-deficient and the ISS models were found, suggesting that they still have different phenotypes after 1 year of treatment, even if the phenotypes of the two groups have become more similar than they were before the start of GH treatment. Thus, we identified protein profiles correlating with the 2-year growth response when data from both children with GH deficiency and ISS were included in the analysis.

The optimal time period needed to detect changes in patterns of protein peaks that may be of utility in predicting long term growth is likely to be different for different variables and in different subset of the population. Previously we have shown that growth over the first year of treatment is a good predictor of long-term growth in response to GH (1-7 years) in prepubertal children [12]. However, it would be of interest to analyze protein expression profiles in relation to growth response to treatment after a period shorter than 1 year. SELDI-TOF was recently used to show that the intensity of several peaks was changed in peripheral blood leukocytes from healthy adults after 4 weeks of GH treatment. However for the majority of the peaks the intensities were reverted to baseline levels after additional 4 weeks of treatment and [37].

Currently, the best models for predicting growth in response to GH treatment are based on early growth data, auxological data of the child and the parents, and hormone levels during the pretreatment year [7,8,11]. However, the data required by such models are not always readily available. Data on early growth in the child, for example, are seldom available. Similarly, growth during the pre-treatment year and/or information on the height of the parents can not always be obtained. In addition, spontaneous GH profiles may not have been assessed, even though it has been shown that a full 24 h GH profile is not necessary [38]. These difficulties highlight the need for improved models that are based only on data that is always available at start of therapy.

During the last decade there has been growing interest in proteomics and systems biology in general. A main focus has been exploring the use of new technology to study complex multigenetic diseases, to predict drug response, to individualize treatment and to discriminate between healthy and diseased individuals [39-41]. We have used SELDI-TOF, a high-throughput technique which is suitable for analyzing large numbers of samples, in order to identify specific protein profiles that are correlated with growth in response to treatment, and to get more insight into GH-dependent regulation of longitudinal growth. The challenge in proteomic analyses of serum is the broad range of expression levels between proteins with low and high abundance [42-44]. In order to partly overcome this problem, we used fractionated serum that was analyzed on different array surfaces in order to detect proteins in a larger area of the proteome.

The reproducibility and reliability of the SELDI-TOF proteomic system have been discussed [45-47]. Concerns about using samples from retrospective studies have been raised as transit time, storage conditions, clotting time and tube type can affect protein profiles [48,49]. However, proper handling of samples can minimize these shortcomings [50]. Our group has established a well-defined protocol for handling and running of samples in proteomic studies [13]. No systematic errors correlated with non-GH-dependent factors or experimental biases such as array or spot number biases were detected. In this study we ran samples on three different surfaces and using three different fractions to cover a larger part of the proteome compared with a single surface and fraction, and in general the most reliable results were found using only the CM10 surfaces. In agreement with other reports, we found a greater number of peaks on the CM10 surface. Moreover, there was a partial overlap between the peaks detected on CM10, IMAC30 and H50, respectively.

Much effort has been put into creating a robust and reliable strategy for the statistical analysis of peak data. Combinations of between-duplicate variation ratio, cross-validated stepwise regression and random permutation tests were performed in order to make certain that the results obtained were robust and reliable.

## Conclusion

In summary, analysis of serum protein expression patterns can be used to identify markers of growth response in short prepubertal children with either GH-deficiency or ISS receiving GH treatment. Our results support previous findings that apolipoproteins and TTR may have a role in GH sensitivity and could be used to predict growth in response to GH treatment in short prepubertal children. The next step will be to test whether or not the incorporation of information on these peaks (either in addition to or in place of existing variables) in our prediction models for prepubertal growth [7,8] will have an additive predictive value in explaining the response to GH treatment.

## Abbreviations

AITT: arginine-insulin tolerance test; Apo A-II: apolipoprotein A-II; CV: coefficient of variation; FT/ICR: Fourier transform ion cyclotron; GH: growth hormone; GHD: GH-deficient; GH_max_: maximum peak of GH secretion; HDL: high-density lipoprotein; IGF-I: insulin-like growth factor I; IGFBP-3: IGF-binding protein 3; ISS: idiopathic short stature; MS: mass spectrometry; LC -MS/MS: liquid chromatography/tandem MS; m/z: mass/charge; LDL: low-density lipoprotein; SDS: standard deviation score; SELDI-TOF-MS: surface-enhanced desorption/ionization time-of-flight mass spectrometry; S/N: signal-to-noise ratio.

## Competing interests

KAW declares that she received an unrestricted research grant from Pharmacia/Pfizer until 2005. AFMN works for Muvara, Multivariate Analysis of Industrial and Research Data Statistical Consultation, The Netherlands.

GH, BA and ZH declare that they have no competing interests.

## Authors' contributions

BA, GH, AFMN, ZH and KAW have all given substantial contribution to conception and design, analysis and interpretation of the data. BA, GH and KAW have designed the experiment and BA has carried out all the SELDI-TOF and additional experiments. AFMN performed most of the statistical analyses, the rest have been performed by BA. GH, BA, AFMN, ZH and KAW have all been involved in drafting the manuscript and have revised it critically for intellectual content.
